# Studying hemispheric lateralization of 4-month-old infants from different language groups through near-infrared spectroscopy-based connectivity

**DOI:** 10.3389/fpsyt.2022.1049719

**Published:** 2022-11-24

**Authors:** Chenyang Gao, Leijin Shu, Ting Li

**Affiliations:** Laboratory of Artificial Intelligence Theranostics, Institute of Biomedical Engineering, Chinese Academy of Medical Sciences and Peking Union Medical College, Tianjin, China

**Keywords:** infant brain network, resting-state, functional near-infrared spectroscopy, functional connectivity, hemispheric lateralization

## Abstract

**Introduction:**

Early monolingual versus bilingual experience affects linguistic and cognitive processes during the first months of life, as well as functional activation patterns. The previous study explored the influence of a bilingual environment in the first months of life on resting-state functional connectivity and reported no significant difference between language groups.

**Methods:**

To further explore the influence of a bilingual environment on brain development function, we used the resting-state functional near-infrared spectroscopy public dataset of the 4-month-old infant group in the sleep state (30 Spanish; 33 Basque; 36 bilingual). Wavelet Transform Coherence, graph theory, and Granger causality methods were performed on the functional connectivity of the frontal lobes.

**Results:**

The results showed that functional connectivity strength was significantly higher in the left hemisphere than that in the right hemisphere in both monolingual and bilingual groups. The graph theoretic analysis showed that the characteristic path length was significantly higher in the left hemisphere than in the right hemisphere for the bilingual infant group. Contrary to the monolingual infant group, the left-to-right direction of information flow was found in the frontal regions of the bilingual infant group in the effective connectivity analysis.

**Discussion:**

The results suggested that the left hemispheric lateralization of functional connectivity in frontal regions is more pronounced in the bilingual group compared to the monolingual group. Furthermore, effective connectivity analysis may be a useful method to investigate the resting-state brain networks of infants.

## Introduction

The human brain is a complex dynamic system that usually behaves as a structurally or functionally interrelated network (1). Studying neural connectivity patterns could provide worthy insights into the working mechanisms of the human brain. The linguistic function influenced brain development by age factors (2). However, the neural mechanisms of bilingual factors based on brain connectivity during early brain development remain unclear. Previous studies showed that early environmental factors including caregiver education level or social and economic status can alter functional brain connectivity (3, 4). A study proposed that the bilingual environment of the infant in the first months of life leads to a series of biochemical events at the microscopic level. This increased the generation of cellular substrates that regulate neuroplasticity and the timing of their synthesis. This may lead to structural changes at the macroscopic level, as manifested by an increase in the size of specific brain language areas and stronger connectivity between brain regions associated with language function (5).

Until now, few studies focused on functional connectivity differences in resting-state brain networks in infants as young as 4-month-old. Based on these findings, the purpose of this study was to compare whether 4-month-old infants growing up in monolingual and bilingual environments exhibit differences in functional connectivity. To answer this question, Blanco B et al. (6) studied resting-state functional connectivity (RSFC) at the group level in 4-month-old infants with functional near-infrared spectroscopy (fNIRS). However, statistical tests showed no differences in RSFC between infants growing up in a monolingual/bilingual background at 4 months of age. We intended to perform a complementary analysis from the perspective of hemispheric lateralization based on their study.

RSFC can be defined as synchronized brain activity between brain regions that function together in supporting functionally relevant sensory and cognitive processes. RSFC can be measured in humans of all ages. It provides a vehicle for neurospecialization throughout the lifespan. Studies showed that RSFC is reliable for accessing language-related networks in clinical settings (7). fNIRS is a promising technology. It allows continuous data acquisition for long periods of time with high temporal sampling rates. In addition, there is a little physical limitation on the participants. As an emerging neuroimaging tool, fNIRS spectroscopy has been successfully used to localize brain activation during cognition and to determine the functional connectivity of resting-state brain activity (8).

MRI studies of adults suggested that long-term exposure to two languages may alter functional connectivity in the brain (9, 10). Studying the RSFC in monolingual and bilingual infants may shed light on the effects of long-term environmental factors (e.g., early bilingual experiences) on the intrinsic properties of functional brain systems.

Based on previous studies of lateralization related to functional brain organization (11–13), and considering the spatial resolution and coverage of the fNIRS setting, it is expected that differences in functional connectivity will appear in regions of the frontal lobe involved in language networks in bilingual infants.

## Materials and methods

### Data acquisition

**Figure 1:**
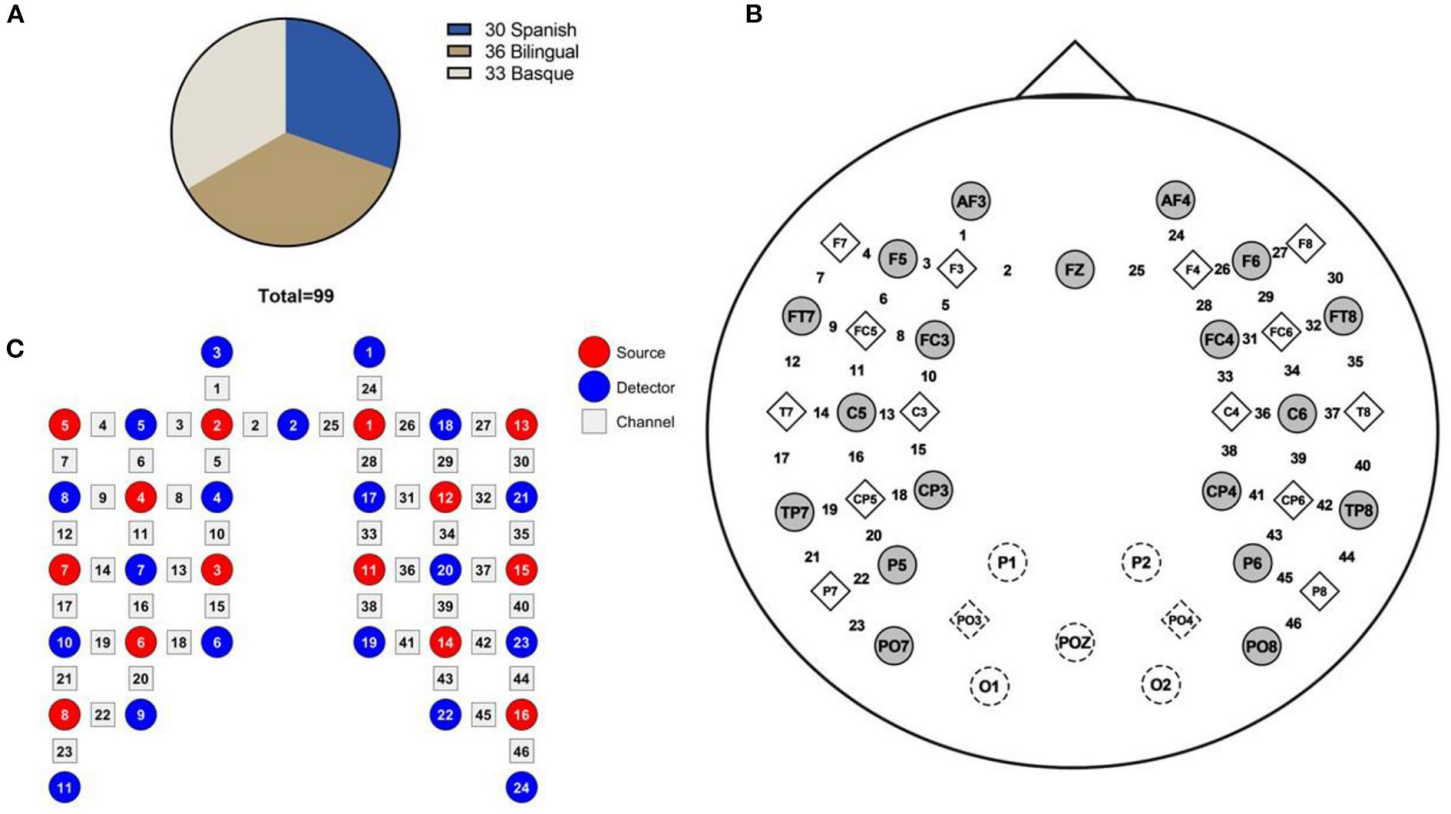
**(A)** Data set consisting of 99 infants, **(B)** fNIRS channel locations on the brain image, and **(C)** Schematic diagram of the source and detector optodes.

The experimental dataset of the study subjects was taken from the Open Science Framework (OSF) website (6). The subject composition of this dataset included Basque monolingual infants (33), Spanish monolingual infants (30), and bilingual (Spanish-Basque) infants (36) with a mean age of 4 months old (Figure 1A). The datasets were recorded during the infant's sleep.

### Pre-processing of data

**Figure 2:**
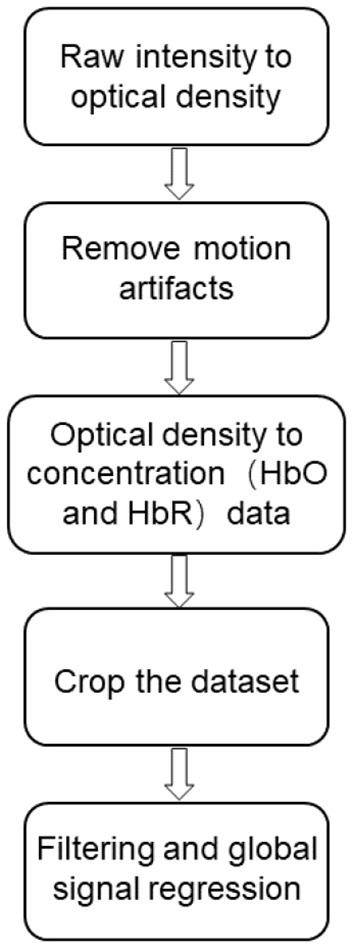
fNIRS data pre-processing flow chart.

The fNIRS signal was pre-processed using the results from the dataset by Blanco et al. (14) after pre-processing, the dataset was pre-processed in MATLAB using internal scripts and third-party functions. The pre-processing flow is shown below (Figure 2).

(1) First, the raw light intensity data are converted into optical density variations (15).
(2) The wavelet-based denoising method proposed by Patel et al. (16, 17) is applied to reduce motion artifacts.
(3) Converting the optical density data into oxyhemoglobin (HbO) and deoxyhemoglobin (HbR) concentration variations by the modified Beer-Lambert law with differential path length factors of 5.3 and 4.2 (18).
(4) All data sets were manually cropped to 5,000 samples (~560 s).
(5) Interference regression models including time-domain filtering and global signal regression were applied to attenuate fNIRS signal contamination due to systematic fluctuations in global sources (e.g., respiration and cardiac pulses) (19–22).

### Analysis of data

To explore differences in brain network connectivity in infants from different language backgrounds. Functional connectivity, effective connectivity, and graph theory analysis were performed on 9-min sleep resting-state fNIRS signals from a group of 4-month-old infants from three language backgrounds.

#### Analysis of the functional connectivity of the bilateral frontal lobes

Functional connectivity is a non-directional method for analyzing brain functional connectivity. It assesses the statistical correlation of activity in different regions of the brain from a functional integration perspective (23). In this study, the functional connectivity of bilateral frontal lobes was analyzed by Wavelet Transform Coherence (WTC) method. The WTC method is a time-frequency analysis method that has been successfully applied to the field of resting-state brain network connectivity (24). It can evaluate the local intercorrelation of two time series. Therefore, it can reveal local phase information that is difficult to find by traditional time series analysis methods (e.g., Fourier transformation) (25). This study used the wavelet transform coherence toolkit developed by Grinsted et al. (download at: http://noc.ac.uk/using-science/crosswavelet-wavelet-coherence). First, the correlation between symmetrical channels in the left and right frontal regions (e.g., 1–24 and 2–25 are corresponding channels) was analyzed using the WTC (Figure 1B). All eight channel pairs (1–24, … 8–31) were located in the left/right frontal regions. Channels 1, 3, and 5 are located in the left frontal middle gyrus. Channel 2 is located in the left frontal superior gyrus. Channels 4, 6, 7, and 8 are located in the left inferior frontal gyrus. Channels 24, 26, and 28 are located in the right middle frontal gyrus. Channel 25 is located in the right superior frontal gyrus. Channels 27, 29, 30, and 31 are located in the left inferior frontal gyrus (Figure 1C). The mean values of coherence value in the 0.02–0.25 Hz band were calculated to represent the strength of the RSFC. We conducted eight one-way ANOVA on the functional connectivity values between different language groups for each of the eight channel pairs. Multiple comparison analysis was performed between different language groups for channel pairs with significant one-way ANOVA. The Fisher-*z* transform was performed on the coherence values before the multiple comparison analysis.

#### Functional connectivity analysis of frontal regions in the ipsilateral hemisphere

Correlation analysis of the time series of HbO signals between any two channels in the left and right hemispheric interfrontal regions (L1–L8, R1–R8) was performed using the WTC analysis, respectively. The average of the coherence values in the 0.02–0.25 Hz band was calculated to characterize the functional connectivity strength within the cerebral hemispheres. After this step, the left and right intra hemispheric frontal functional connectivity matrices were obtained. The average intra hemispheric functional connectivity matrix based on HbO signals between subjects in each of the three language background conditions was plotted separately. Each element of the functional connectivity matrix is the correlation value between the two channels represented by its horizontal and vertical coordinates. We first performed a two-way ANOVA on the unilateral functional connectivity values of left/right frontal regions for different language groups. Then differences in unilateral functional connectivity values between left and right frontal regions in monolingual and bilingual background groups were analyzed using *post-hoc t*-tests. The Fisher-*z* transform was performed on the coherence values before the *post-hoc t*-tests.

#### Small-world characterization in the ipsilateral hemisphere

First, we traversed the all channels in the left and right cerebral hemispheres to calculate all the two wavelet coherence value sequences of the HbO signal and obtain the adjacency matrix. The i-th row of the adjacency matrix represents the correlation value between channel i and other channels, which represents the strength of functional connectivity. Then the statistical properties of the small-world network are calculated based on the adjacency matrix. The clustering coefficient is analyzed to evaluate the degree of node aggregation and the characteristic path length is analyzed to evaluate the global characteristics of the network. The distance d_ij_ between node i and node j in the network is the number of shortest path edges connecting the two nodes. The characteristic path length L_mean_ is the average of the shortest paths between any two points in the network, where *N* is the number of nodes of the network., i.e.,


(1) $$\begin{array}{l} L_{mean}=\frac{1}{\frac{1}{2}N\left(N-1\right)}\underset{0<i<j}{\sum }d_{ij} \end{array}$$

The clustering coefficient is used to characterize the clustering properties of the network nodes. Assuming that node i in the network is associated with k_i_ edges, it is known that between these k_i_ nodes, there are at most k_i_^*^ (k_i_-1)/2 edges, and the actual number of edges existing between these k_i_ nodes is E_I_. The ratio of the actual number of edges existing between these k_i_ nodes, E_I_, to the total maximum possible number of edges, k_i_^*^(k_i_-1)/2, is defined as the clustering coefficient of node i, C_i_, i.e.,


(2) $$\begin{array}{l} C_{i}=\frac{2E_{I}}{k_{i}\left(k_{i}-1\right)} \end{array}$$

The clustering coefficients of all nodes in the network C_i_ are averaged to obtain the clustering coefficients of the whole network C_Mean_, where *N* is the number of nodes of the network, i.e.,


(3) $$\begin{array}{l} C_{Mean}=\frac{1}{N}\overset{N}{\underset{i=1}{\sum }}C_{i} \end{array}$$

In addition, the brain function network studied in this paper is a weightless network, in which there are two states between any two nodes, connected or unconnected. To distinguish between connected and unconnected, we need to define a threshold *T*. When the connection strength between two nodes is greater than or equal to this threshold, the two nodes are considered to be connected; when it is less than this threshold, the two nodes are considered to be unconnected. In the fNIRS signal, once the threshold is set, the brain network can be built to study the small-world properties. Among them, it should be noted that if the threshold is set too high, it will lead to too many isolated nodes in the network and may miss some important information; if it is set too low, the connectivity density is too large and it will cause too many pseudo-connections. The range of the threshold *T* is determined by the conditions of the average node degree and sparsity of the network. It is required that all subjects satisfy the following two conditions (26): First, the average node degree is greater than twice the natural logarithm of the number of nodes *N*, in this paper *N* = 23. Therefore, for all subjects, the average node degree of the network must be no <6. Second, the network density (the ratio of the number of connected edges actually present in the network to the maximum number of possible edges in the network) is <50%. The threshold *T* to satisfy the above condition ranges from 0.51 < *T* < 0.71. To verify the robustness of the graph theory analysis results, we set the threshold *T* to range from 0.51 to 0.71, step size 0.01. Finally, we averaged the 21 C and L values for each subject separately (27).

#### Analysis of the effective connectivity of the bilateral frontal lobes

Effective connectivity analysis is a directional method of brain connectivity analysis. The effective connectivity between different brain regions describes the causal relationship between the interactions of different brain regions. The analysis results can reflect the information flow transmission between different brain regions. In this study, the effective connectivity of the bilateral frontal lobes was analyzed using the Granger Causality (GC) mathematical model (23, 28). The calculation of the GC analysis was based on an autoregressive model. In the process of analyzing two data series using GC analysis. Signal A is said to “Granger cause” signal B if information from the past of A helps to better predict B than only considering information from the past of B (28).

In order to explore the hemispheric lateralization properties of infants under different language background conditions. This study used GC analysis for the corresponding channels between the frontal lobes of the left and right hemispheres (e.g., 2–25 and 5–28 are corresponding channels). The GC values were calculated for the corresponding channel left-to-right and right-to-left in bilateral frontal lobes, respectively. The GC value from the left-to-right direction minus the GC value from the right-to-left direction was defined as the difference of influence (DOI), which indicates the net causality from the left-to-right direction (29). The DOI values were calculated for each channel pair within the left/right frontal region based on the HbO signals. For example, channel 1 in the left frontal region was paired with channel 24 in the right frontal region. Then we averaged the DOI values of all channel pairs as the value of effective connectivity for the subject (27). DOI values were calculated separately for each language background condition.

In this study, we used the Granger causal connectivity analysis MATLAB toolkit (Granger causal connectivity analysis, GCCA) developed by the University of Sussex to analyze the RSFC (29). This toolkit includes data pre-processing, data stationary check, model validity and consistency verification, and DOI calculation. The choice of the lag period in the GC model affects the Granger output results. Therefore, in order to verify the robustness of the effective connectivity analysis (28), multiple lag period parameters (2nd order, 3rd order, 4th order, and 5th order) were carried out with the GC method. We first performed four one-way ANOVAs on the DOI values between different language groups at four orders (2, 3, 4, and 5). Multiple comparison analyzes were conducted for orders with significant one-way ANOVA results, and FDR correction was applied to the statistics.

## Results

### Results of functional connectivity analysis of bilateral frontal lobes

**Figure 3:**
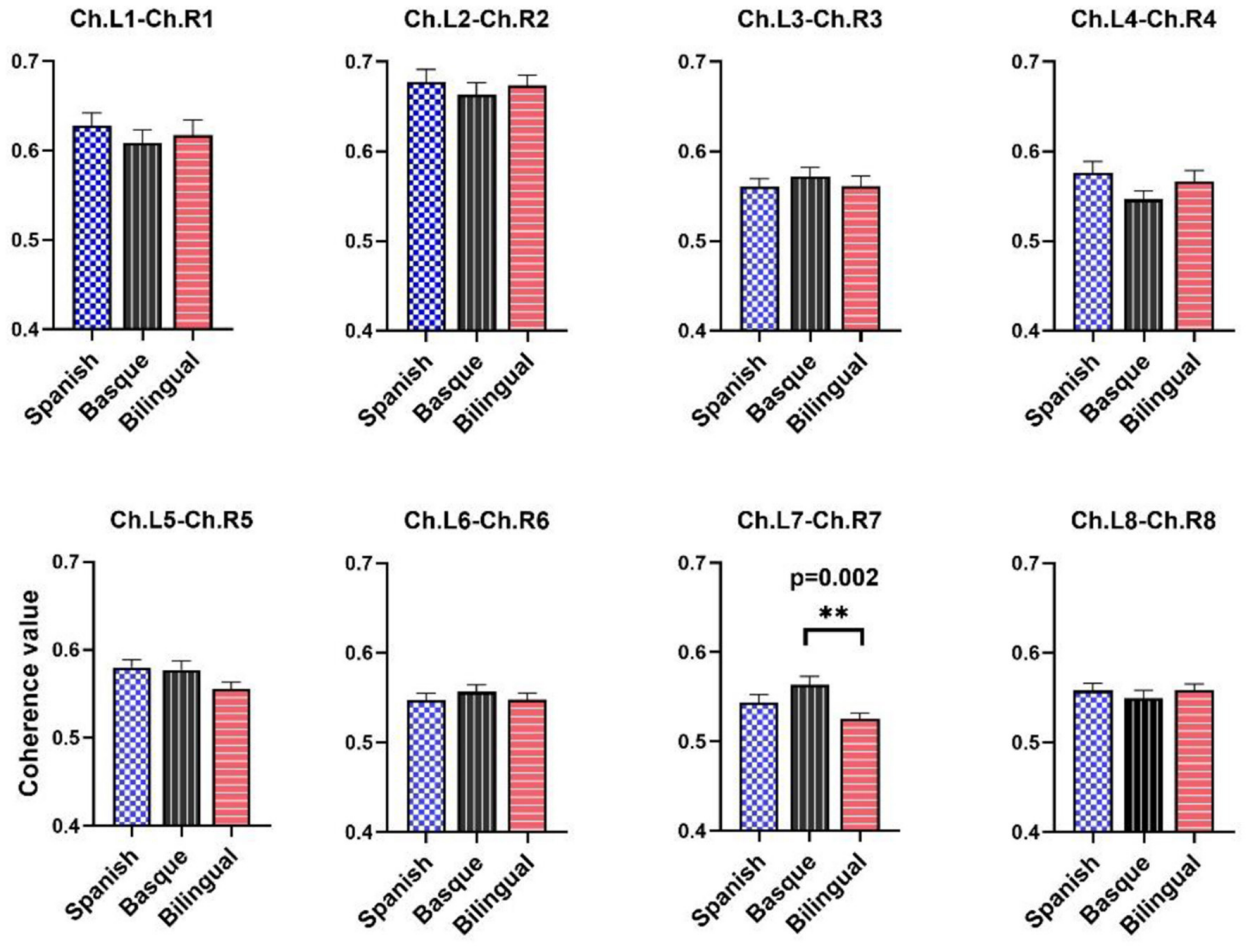
Average coherence values between left and right frontal symmetric channels. The data are expressed as mean values SE. The black asterisks indicate *p*-value and FDR correction.

Statistical tests showed differences in resting-state brain functional connectivity between infants with different language backgrounds (Figure 3). Only the results of the one-way ANOVA of the channel pair “L7–R7” on the functional connectivity values between the three language groups were significant [*F* (2, 87) = 5.813, *p* = 0.0043]. The results of the multiple comparison analysis between the functional connectivity values of the three language groups showed that the Basque group was significantly higher than the bilingual group [*t* (87) = 3.409, *p* = 0.0021]. No significant differences were found between the Basque group and the Spanish group [*t* (87) = 1.744, *p* = 0.0696]. There was no significant difference between the Spanish-speaking group and the bilingual group either [*t* (87) = 1.665, *p* = 0.0696]. The results of the multiple comparison analysis were corrected using a false discovery rate (FDR) correction to control for the expected percentage of false “discoveries.” Unfortunately, no significant differences were observed in the mean strength of functional connectivity of all eight channel pairs in the frontal lobe.

### Results of functional connectivity analysis of ipsilateral frontal lobes

**Figure 4:**
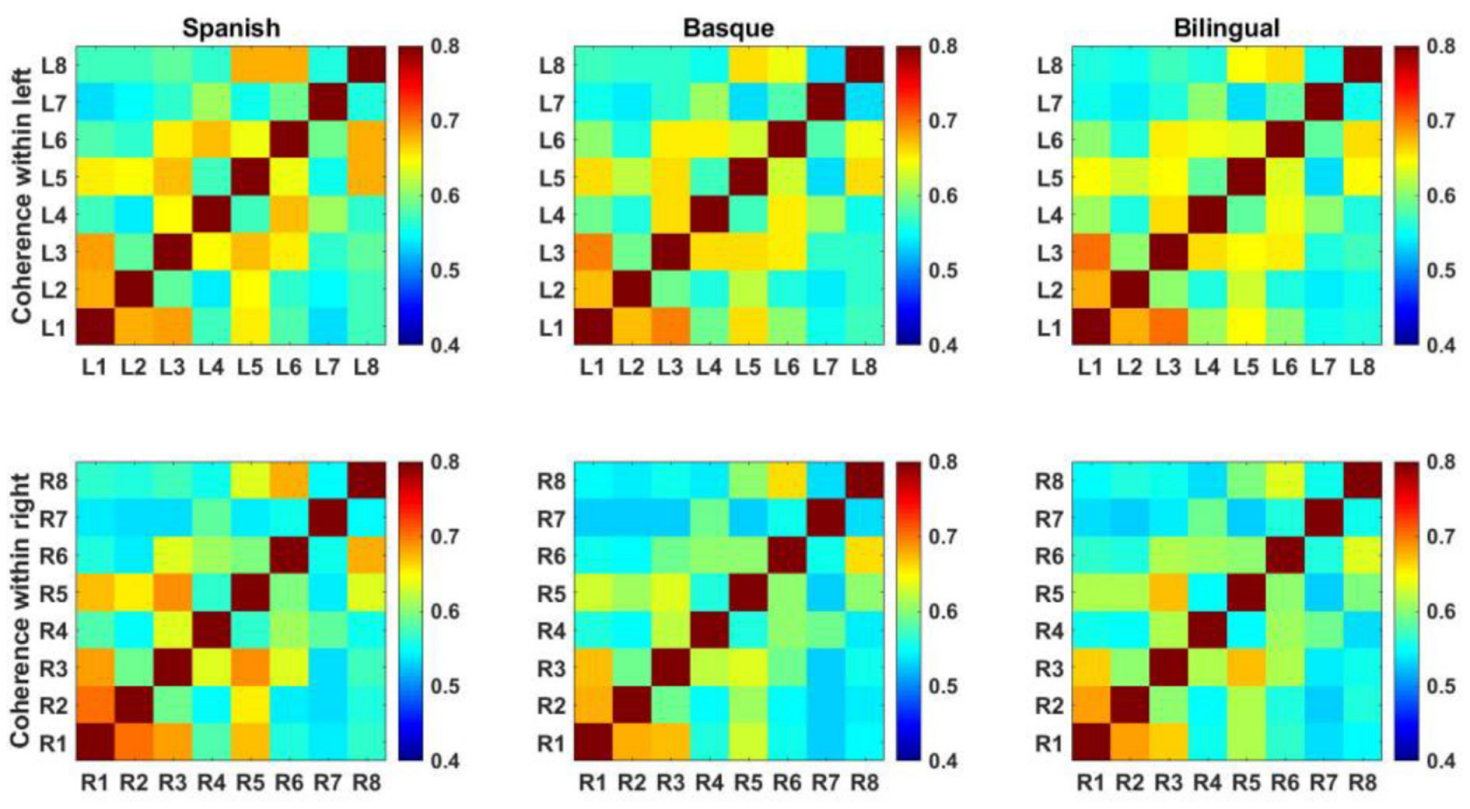
Intrahemispheric functional connectivity matrix based on oxyhemoglobin.

Each element of the functional connectivity matrix is the correlation value between the two channels represented by the horizontal and vertical coordinates (Figure 4).

**Figure 5:**
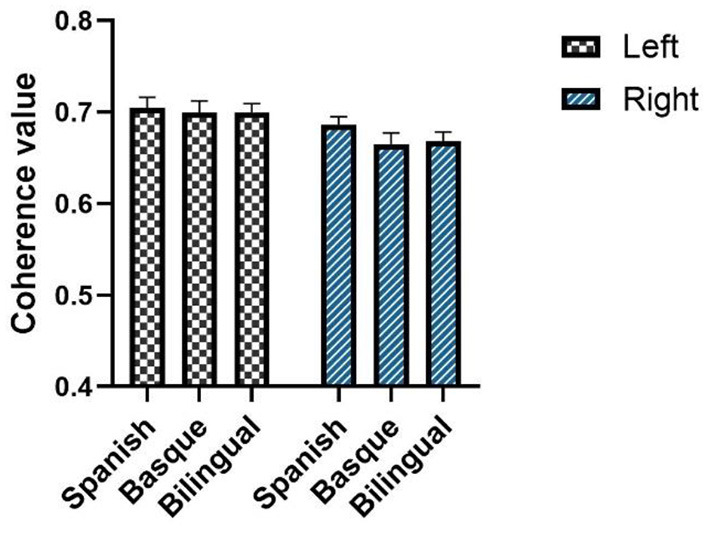
Average of intrahemispheric functional connectivity based on oxyhemoglobin. The data are expressed as mean values SE. The black asterisks indicate *p*-value and FDR correction.

The mean values of the elements in the functional connectivity matrix were calculated to characterize the functional connectivity strength of the frontal lobes on both sides. The elements on the sub diagonal of the functional connectivity matrix showed the autocorrelation results for each fNIRS channel with a value of 1. These values were not involved in the process of calculating the mean of the connectivity matrix. A two-way ANOVA showed that the interaction term was not significant [*F* (2, 174) = 0.2888, *p* = 0.7495] (Figure 5).

### Results of small world characterization in the hemisphere

**Figure 6:**
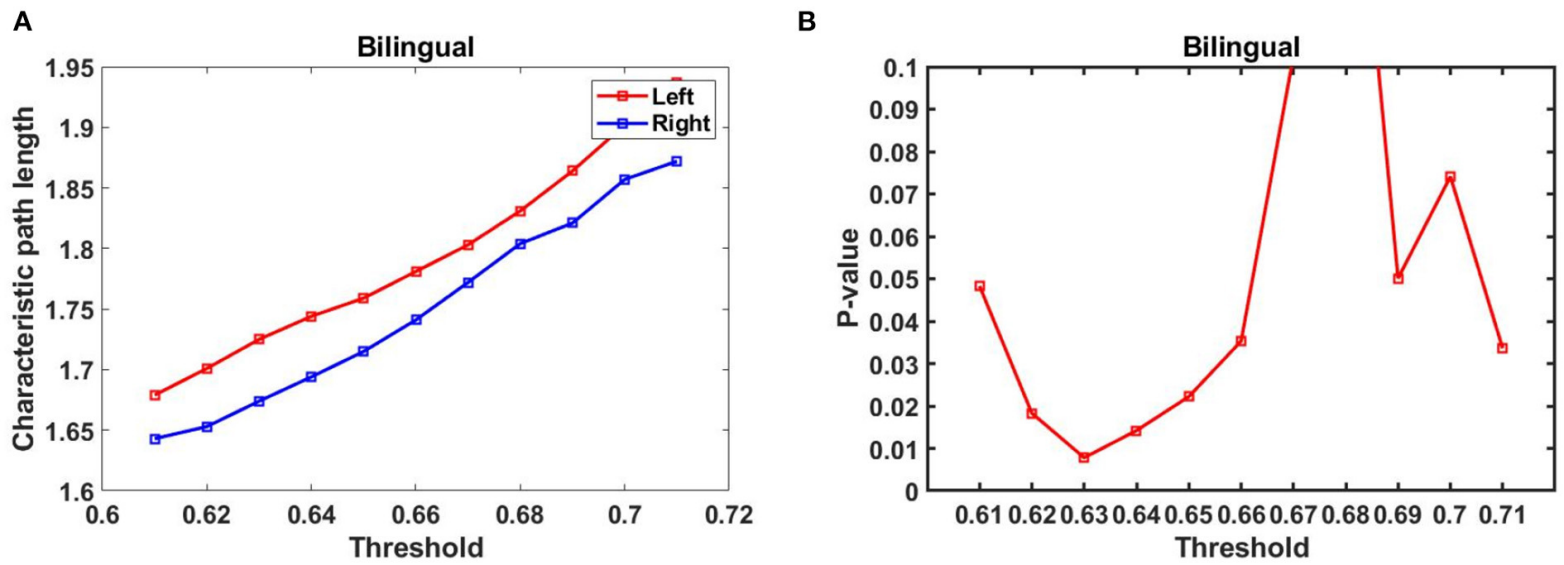
**(A)** L values in the threshold range of the bilingual group, **(B)** *p*-values in the threshold range for bilingual groups (FDR correction).

Based on the functional connectivity matrix of the brain network. The average L and C values based on functional connectivity for three groups in the left and right frontal were displayed in Table 1, and the asterisks (*) represent significant differences (*p* < 0.05, FDR correction). Only the bilingual infant group showed significant differences in L value [*t* (29) = 2.497, *p* = 0.0185], which indicated that infants in the bilingual group had more optimal left hemisphere lateralization compared to infants in the monolingual group. No significant differences were found in the monolingual group. We noticed that part of the *p*-values of the bilingual group were significant when 0.61 < *T* < 0.71 (Figures 6A,B).

**Table 1:** Characteristic path length based on functional connectivity, the asterisks (*) represent significant differences (p < 0.05, FDR correction).

	**Bilingual**	**Spanish**	**Basque**
L-left	1.6890*	1.6674	1.6935
L-right	1.6665*	1.6783	1.6781
C-left	0.5607	0.5461	0.5576
C-right	0.5498	0.5401	0.5384

### Results of effective connectivity analysis of bilateral frontal lobes

**Figure 7:**
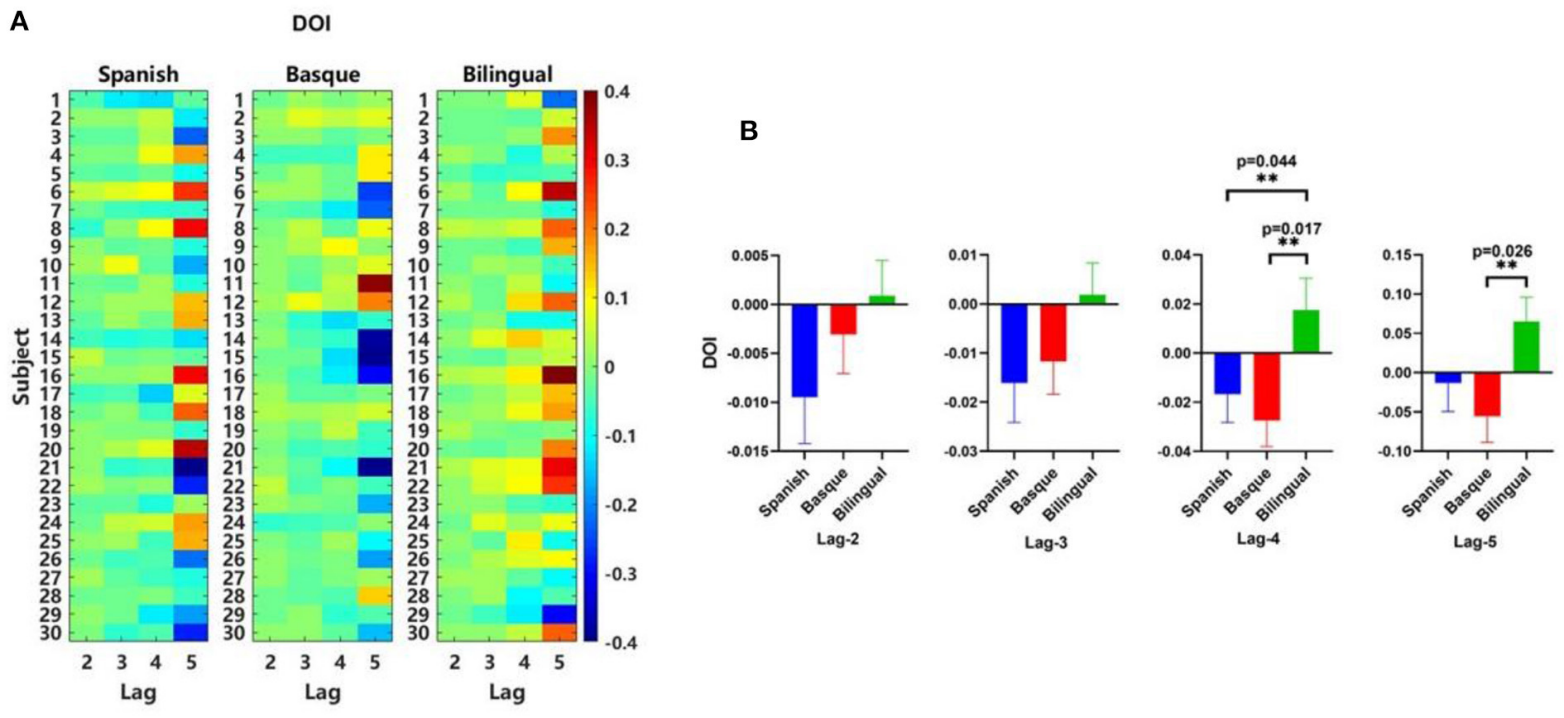
**(A)** Matrix of DOI values, **(B)** Mean DOI values calculated based on oxyhemoglobin signals.

We noticed that for all lag period parameters, DOI values were negative in the left and right frontal lobes of the monolingual group, while DOI values were positive in the frontal lobes of the bilingual group (Figure 7A). The results showed that the direction of effective connectivity between the left and right frontal lobes of bilingual infants was opposite to monolingual infants (Figure 7B). One-way ANOVA results for DOI values for different language groups at different orders showed that orders 4 [*F* (2, 87) = 4.031, *p* = 0.0212] and 5 [*F* (2, 87) = 3.348, *p* = 0.0397] were significant. The results of the multiple comparison analysis of DOI values between the different language groups were as follows. Lag 4: There was a significant difference between the Spanish group and the bilingual group [*t* (87) = 2.068, *p* = 0.0437]. Significant differences were also observed between the Basque group and the bilingual group [*t* (87) = 2.719, *p* = 0.0166]. No significant difference between the Spanish group and the Basque group [*t* (87) = 0.6511, *p* = 0.3617]. Lag 5: There was a significant difference between the Basque group and the bilingual group [*t* (87) = 2.550, *p* = 0.0263]. There were no significant differences between the Spanish and bilingual groups [*t* (87) = 1.657, *p* = 0.1063]. No significant differences between the Spanish and the Basque groups either [*t* (87) = 0.8931, *p* = 0.2620] (Figure 7B). The results of the two multiple comparison analyzes were corrected using a false discovery rate (FDR) correction to control for the expected percentage of false “discoveries.”

## Discussion

This study aimed to explore the hemispheric lateralization properties of RSFC in 4-month-old infants based on fNIRS. Functional connectivity in frontal regions of the left and right hemispheres was measured using WTC analysis and GC analysis. The hemispheric lateralization properties of functional connectivity were verified using graph-theoretic analysis.

Several research groups reported the use of resting-state functional near-infrared spectroscopy (R-fNIRS) for the assessment of functional connectivity (8, 29, 30). The results have shown that RSFC analysis based on R-fNIRS data is valid and reliable for studying brain function in healthy and diseased populations. Hemodynamic regulation in the resting state and the connections within and between functional networks can be measured efficiently by optical methods. In addition, gender differences in brain networks in visual working memory have been previously investigated using GC methods (27). The above demonstrated that the analysis methods in this study are reasonable and valid.

The results of the unilateral functional connectivity analysis showed left hemispheric lateralization of functional connectivity in frontal regions in both the bilingual and Basque infant groups. The results of the graph theory analysis confirmed the finding that the left hemisphere lateralization of functional connectivity was more pronounced in bilingual infants. Significant differences in the mean characteristic path lengths between the left and right hemispheres were also shown in the results of the graph theory analysis. This may imply that the intrinsic functional organization of infants growing up in a bilingual environment is more shaped. We will further conduct a more detailed analysis later using other parameters of the small-world characteristics and other analysis methods. In this way, we will find enough evidence to make the conclusions more convincing. Previous studies have shown that frontal and temporal brain regions associated with language function have significant left hemisphere lateralization in adult and adolescent groups (9, 31, 32). This confirmed the reliability of our findings. No significant differences were found in the group-level analysis of bilateral and unilateral functional connectivity, which was generally consistent with the findings derived by Blanco et al. (6). However, there were significant differences in functional connectivity strength between language groups in channel pair 7–30, which is located in the inferior frontal gyrus. This may indicated a key role of the inferior frontal gyrus in the development of language function in infants, and we will follow up with the further analysis of the inferior frontal gyrus language center.

The results of the effective connectivity analysis showed that the direction of information flow in the frontal regions of the bilingual infant group was from left to right, whereas the direction of information flow in the monolingual infant group was from right to left. In addition, the average brain connectivity between the bilingual and monolingual infant groups was more significantly different in the effective connectivity analysis than in the functional connectivity analysis. Medvedev et al. (33) have shown that the direction of information flow in effective connectivity analysis is related to the dominant center of the brain in the resting state. This also laterally confirmed the more significant left hemispheric lateralization of brain functional connectivity in frontal regions of bilingual infants. Based on the above findings, we concluded that the effective connectivity analysis could be used as a complement to the resting-state brain network analysis in the present study.

We expected that the findings of this paper can be used as a reference for the study of the lateralization of brain functional networks. We will also further improve the study, such as studying the differences in the lateralization of brain networks in different age groups of monolingual infants and bilingual infants. Furthermore, we will verify and supplement the conclusions of this study, and provide a more comprehensive guide for the application of fNIRS spectroscopy in the analysis of resting-state brain functional connectivity.

## Conclusion

In this study, we found that the functional brain connectivity of infants growing up in a bilingual environment had more significant left hemisphere lateralization properties compared to those growing up in a monolingual environment. Furthermore, effective connectivity analysis can complement the results of resting-state brain network analysis.

## Data availability statement

Publicly available datasets were analyzed in this study. This data can be found here: https://doi.org/10.17605/OSF.IO/7FZKM.

## Author contributions

CG: writing-original draft, conceptualization, formal analysis, and data curation. LS: conceptualization, data curation, and formal analysis. TL: conceptualization, writing—review and editing, and funding acquisition. All authors contributed to the article and approved the submitted version.

## Funding

This study was funded by National Natural Science Foundation of China (No. 81971660), Chinese Academy of Medical Science Health Innovation Project (2021-I2M-042 and 2021-I2M-058), Sichuan Science and Technology Program (No. 2021YFH0004), Tianjin Outstanding Youth Fund Project (No. 20JCJQIC00230), Program of Chinese Institute for Brain Research in Beijing (2020-NKX-XM-14), Basic Research Program for Beijing-Tianjin-Hebei Coordination [19JCZDJC65500(Z)], and Fundamental Research Funds for the Central University (No. 3332019101).

## Conflict of interest

The authors declare that the research was conducted in the absence of any commercial or financial relationships that could be construed as a potential conflict of interest.

## Publisher's note

All claims expressed in this article are solely those of the authors and do not necessarily represent those of their affiliated organizations, or those of the publisher, the editors and the reviewers. Any product that may be evaluated in this article, or claim that may be made by its manufacturer, is not guaranteed or endorsed by the publisher.
